# Persistent neuropsychiatric dysfunction following chronic tiletamine-containing e-cigarette use: a case report

**DOI:** 10.3389/fpsyt.2026.1848688

**Published:** 2026-06-19

**Authors:** Liu Liu, Qi Zhou, Jinhong Chen

**Affiliations:** 1 The School of Clinical Medicine, Hunan University of Chinese Medicine, Changsha, Hunan, China; 2 Department of Sleep Disorders and Neurosis, Hunan Institute of Mental Health, The Second People’s Hospital of Hunan Province (Brain Hospital of Hunan Province), Hunan University of Chinese Medicine, Changsha, Hunan, China

**Keywords:** case report, craving, e-cigarette, neuropsychiatric dysfunction, tiletamine

## Abstract

Tiletamine is a dissociative anesthetic used in veterinary medicine and has recently emerged as a potential illicit additive in e-cigarettes after regulation of etomidate. However, reports of chronic tiletamine-containing e-cigarette use remain scarce. We report the case of a 22-year-old man who developed progressive gait instability, dysarthria, choking on drinking, hand tremor, emotional instability, and marked craving after chronic use of tiletamine-containing e-cigarettes. Brain MRI and routine electromyography showed no definite structural abnormalities, whereas electroencephalography and blink reflex testing suggested functional brain involvement. After discontinuation of tiletamine-containing e-cigarettes and comprehensive treatment, his neurological symptoms, emotional symptoms, and craving gradually improved, with sustained improvement at 1-month follow-up. This case suggests that chronic tiletamine abuse via e-cigarettes may cause persistent neuropsychiatric dysfunction beyond the acute intoxication pattern described in previous reports and may also be accompanied by dependence-related features.

## 1 Introduction

New psychoactive substances (NPS) pose increasing challenges for clinical diagnosis and public health because of their rapidly changing composition, concealed distribution, and limited toxicological characterization (1, 2). E-cigarettes, initially developed as nicotine-delivery systems, have increasingly been used as adaptable and concealed platforms for substances beyond nicotine, including illicit or adulterated psychoactive compounds (3).

In China, etomidate-containing e-cigarettes were previously reported as an important form of adulterated vaping product (4). After etomidate was included in China’s Class II psychotropic substances catalog on October 1, 2023, the active ingredients in adulterated e-cigarettes showed a substitution trend, with tiletamine, ethyl fluoroketamine, and other substances gradually becoming newly added illicit components (5). Tiletamine is a dissociative anesthetic and phencyclidine derivative that is not approved for human use in either China or the United States and is mainly used in veterinary practice, commonly in combination with zolazepam as Telazol or Zoletil (6–9).

Previous reports of tiletamine or tiletamine/zolazepam misuse have mainly described acute intoxication or emergency presentations, such as involuntary movements, severe tremor, altered consciousness, respiratory depression, hypotension, and fatal poisoning (5, 7, 10–14). In addition, evidence from young recreational ketamine users suggests that repeated non-medical exposure to a related NMDA receptor antagonist may be associated with psychotic-like experiences and impaired psychosocial functioning (15). However, reports of chronic tiletamine-containing e-cigarette use remain scarce, especially those describing persistent neuropsychiatric dysfunction and dependence-related features.

Here, we report a young man who developed progressive gait instability, dysarthria, choking on drinking, hand tremor, emotional instability, and marked craving after chronic use of suspected tiletamine-containing e-cigarettes. This case suggests that suspected chronic abuse of tiletamine-containing e-cigarettes may not only present as acute intoxication, but may also be associated with persistent neuropsychiatric dysfunction accompanied by dependence-related features.

## 2 Case presentation

A 22-year-old man began using etomidate-containing e-cigarettes in October 2024. During this period, he also had brief exposure to flutomidate-containing e-cigarettes for approximately 1 week, after which his main exposure shifted to tiletamine-containing e-cigarettes. Since August 2025, he had repeatedly used tiletamine-containing e-cigarettes, averaging more than 10 cartridges per day. The patient stated that he was aware of this claimed ingredient at the time of purchase, although the actual chemical composition of the cartridges was not analytically confirmed.He reported that inhalation produced a subjective feeling of being “clearer” and “comfortable.” Beginning in October 2025, he gradually developed gait instability, fatigue, slurred speech, choking on drinking, and hand tremor, accompanied by emotional instability, irritability, agitation, and social withdrawal. He denied hallucinations, delusions, or other psychotic symptoms. As these symptoms progressively worsened, he stopped using tiletamine-containing e-cigarettes in November 2025. However, his symptoms persisted after cessation, and he continued to experience marked morning craving. He subsequently received traditional Chinese medicine and acupuncture treatment at another hospital, with poor perceived benefit. On January 6, 2026, he presented to the outpatient clinic of the Addiction Medicine Center, rather than the emergency department, because of repeated e-cigarette use for more than 1 year and a nearly 3-month history of gradually developing hand tremor, slurred speech, and unsteady walking. He was subsequently admitted to the inpatient ward for further diagnostic evaluation and treatment.

At admission, rating scales showed a PHQ-9 score of 15, indicating moderate-to-severe depressive symptoms; a GAD-7 score of 9, indicating mild anxiety symptoms; a SARA score of 20, indicating prominent ataxic manifestations; and a visual analog scale (VAS) score of 6 for craving for tiletamine-containing e-cigarettes, indicating moderate craving.These assessments were performed outside the acute intoxication phase. Because the patient presented with persistent neurological symptoms, affective symptoms, and craving after cessation, the PHQ-9, GAD-7, SARA, and craving visual analog scale (VAS) were used to quantify baseline depressive symptoms, anxiety symptoms, ataxia-like manifestations, and craving severity, respectively, and to monitor changes during hospitalization and follow-up.

On admission, his temperature was 36.2 °C, pulse 85 beats/min, respiratory rate 20 breaths/min, and blood pressure 135/84 mmHg. Cardiopulmonary and abdominal examinations were unremarkable. Neurological examination showed clear consciousness, dysarthria, a diminished pharyngeal reflex, and uvular deviation to the left. Palmomental reflexes were questionably positive bilaterally, more pronounced on the left. Muscle strength and tone were normal in all four limbs. Superficial and deep sensation were symmetrical and normal bilaterally. Babinski signs were negative bilaterally. Finger-to-nose testing, rapid alternating movements, and heel-knee-shin testing were impaired. Romberg testing was positive, and gait was broad-based. The neck was supple, and Kernig’s sign was negative. The patient denied current use of other illicit substances or definite co-ingestions at presentation. He also reported no known prior diagnosis of a psychotic disorder. However, no urine or blood toxicological screening for tiletamine or other common psychoactive substances/new psychoactive substances was performed. Therefore, other co-exposures or adulterants could not be excluded based on toxicological testing.

Mental status examination showed that the patient was neatly dressed, cooperative, and fully conscious, with intact orientation to time, place, and person. No illusions, perceptual distortions, or hallucinations were observed. Thought processes were coherent and generally logical, with no obvious pathological thought content elicited. Attention was relatively preserved. Gross intellectual functioning was normal, while comprehension, judgment, and calculation were fair. Affect was generally appropriate, but mood was unstable, and he was irritable; he reported feeling upset, tense, and anxious. Volitional activity was increased. He demonstrated strong craving for tiletamine-containing e-cigarettes and had limited insight.

**Figure 1:**
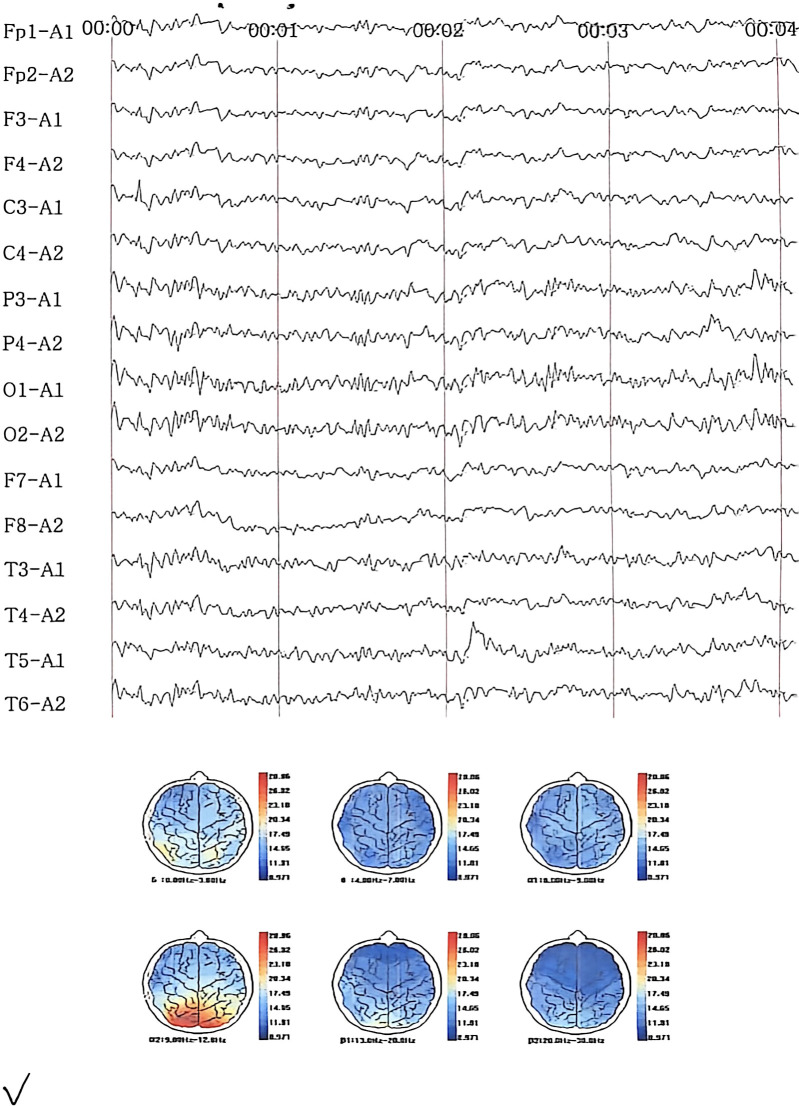
Electroencephalography (EEG) and brain topography findings. EEG/brain topography showed mildly abnormal background activity with beta activity predominance. No typical epileptiform discharges were observed.

**Figure 2:**
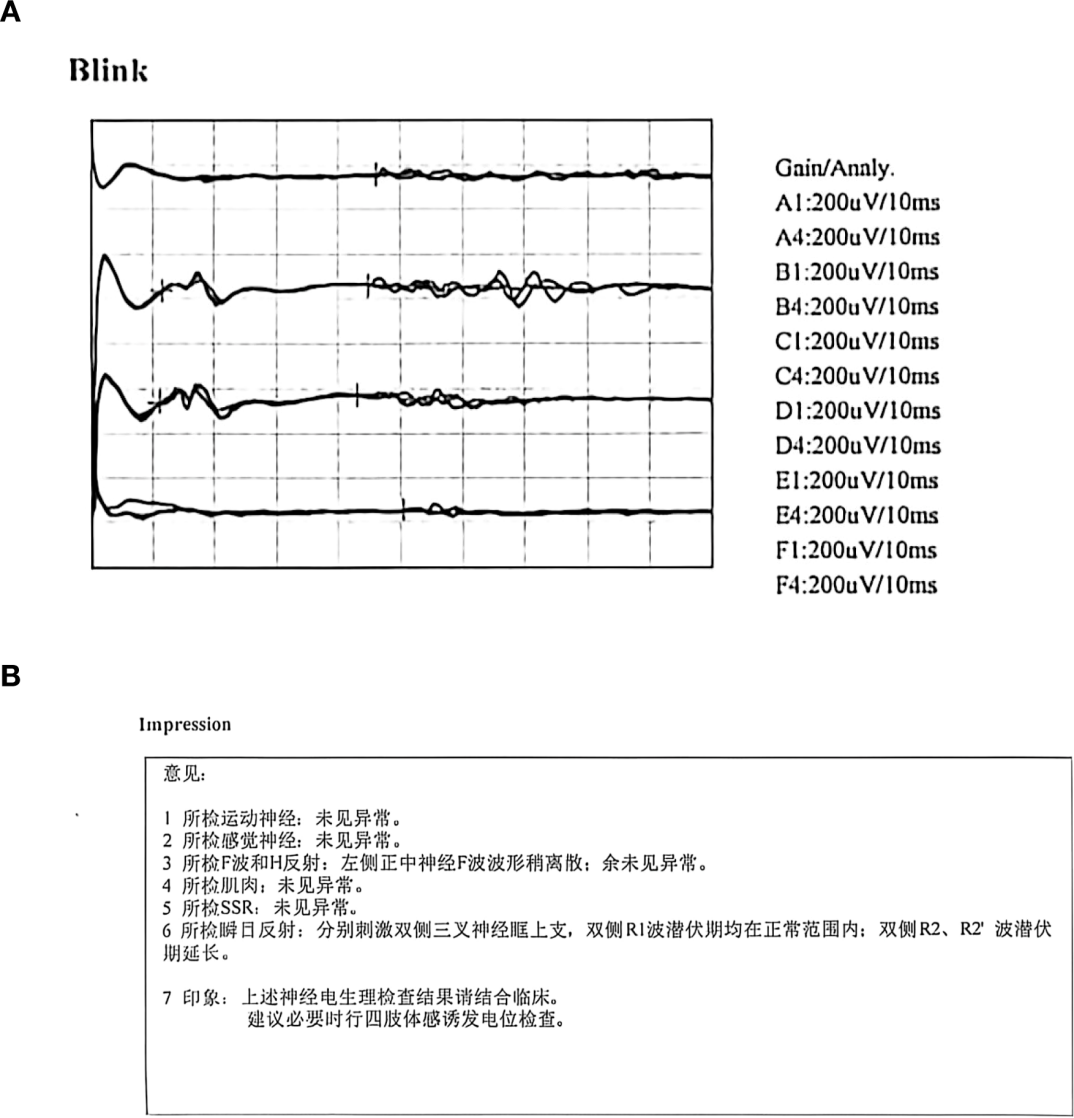
Blink reflex testing findings. **(A)** Representative blink reflex waveforms. **(B)** Neurophysiological report impression showing normal bilateral R1 latencies and prolonged bilateral R2 and R2′ latencies, suggesting delayed late blink reflex responses.

Laboratory, imaging, and neurophysiological examinations were performed after admission. Laboratory tests revealed mild liver dysfunction, with elevated alanine aminotransferase (56.30 U/L) and total bile acids (17.30 μmol/L), as well as elevated homocysteine (18.80 μmol/L). Routine blood testing was essentially normal except for mildly increased basophil and monocyte counts. Myocardial enzymes, myoglobin, troponin I, renal function, electrolytes, coagulation function, blood ammonia, and lactate were all within normal limits. Hepatitis B surface antigen, hepatitis C antibody, treponemal antibody, and HIV antibody were negative. Brain MRI, including DWI, showed no obvious abnormalities. EEG was performed to evaluate possible central nervous system involvement and to exclude epileptiform activity, given the patient’s persistent neurological symptoms.Electroencephalography/brain topography showed mild abnormalities, characterized by a mildly abnormal background with beta activity predominance and no typical epileptiform discharges(**Figure 1**). No clinical seizure activity was reported or observed. Electromyography revealed no clear abnormalities in the examined motor nerves, sensory nerves, or muscles, except for slightly dispersed F-wave morphology in the left median nerve and prolonged bilateral R2 and R2′ latencies on blink reflex testing (**Figures 2A,B**). Abdominal and urinary ultrasonography, as well as bilateral carotid ultrasonography, were unremarkable.The patient’s relevant symptoms, physical examination findings, laboratory values, imaging findings, and neurophysiological examination results are summarized in **Table 1**.

**Table 1:** Summary of relevant symptoms, physical examination, laboratory, imaging, and neurophysiological findings.

Category	Findings
Main symptoms	Repeated e-cigarette use for more than 1 year; nearly 3-month history of gradually developing hand tremor, slurred speech, unsteady walking, fatigue, and choking on drinking; emotional instability, irritability, agitation, social withdrawal, and marked morning craving after cessation; no hallucinations or delusions were reported
Physical and neurological examination	Vital signs were stable. Cardiopulmonary and abdominal examinations were unremarkable. Neurological examination showed dysarthria, diminished pharyngeal reflex, uvular deviation to the left, questionable bilateral palmomental reflexes, impaired coordination tests, positive Romberg test, and broad-based gait. Muscle strength, muscle tone, and sensation were normal, and Babinski signs were negative bilaterally
Laboratory findings	ALT 56.3 U/L, total bile acids 17.3 μmol/L, and homocysteine 18.8 μmol/L. HBsAg, HCV antibody, treponemal antibody, and HIV antibody were negative. Other routine laboratory tests were largely unremarkable.
Imaging findings	Brain MRI, including DWI sequences, showed no obvious abnormalities. Abdominal, urinary, and bilateral carotid ultrasonography were unremarkable.
Neurophysiological examinations	EEG showed mildly abnormal background activity with beta activity predominance, without typical epileptiform discharges. Electromyography showed no clear motor, sensory, or muscle abnormalities, with slightly dispersed F-wave morphology in the left median nerve. Blink reflex testing showed prolonged bilateral R2 and R2′ latencies.

Overall, there was no clear evidence of structural brain injury or organic peripheral nerve or muscle damage, although there were indications of functional brain abnormalities and possible involvement of brainstem-related reflex pathways. Based on the long-term exposure history, clinical manifestations, and ancillary findings, the patient was clinically considered to have persistent neuropsychiatric dysfunction associated with chronic tiletamine-containing e-cigarette use, accompanied by ataxia-like manifestations and dependence-related features.

### Hospital course and follow-up

During hospitalization,the patient received comprehensive supportive treatment after discontinuation of tiletamine-containing e-cigarettes. For neurological symptoms including dysarthria, gait instability, and limb tremor, neurotrophic and neurorestorative treatments were administered, including acupoint injection of vitamin B1 and intramuscular mecobalamin. Following consultation with the neurology department, high-dose vitamin B complex and idebenone were added. Meanwhile, for emotional instability and anxiety, symptomatic psychotropic medications were administered. Magnesium valproate sustained-release tablets were used as a mood stabilizer, initially at 0.25 g every 12 hours and later adjusted to 0.25 g three times daily. Anxiety symptoms were managed with buspirone hydrochloride tablets, initially at 5 mg three times daily and later increased to 10 mg three times daily, together with short-term oxazepam, initially at 15 mg every 12 hours and later adjusted to 15 mg nightly. Acupuncture and traditional Chinese medicine were also provided as adjunctive therapies based on syndrome differentiation.

After comprehensive treatment, tremor, dysarthria, and gait abnormalities gradually improved, mood stabilized, and craving decreased. At discharge, rating scales showed improvement: the SARA score decreased from 20 to 10, the PHQ-9 score from 15 to 6, the GAD-7 score from 9 to 3, and the craving VAS score for tiletamine-containing e-cigarettes from 6 to 0, indicating marked improvement in neurological symptoms, emotional symptoms, and craving compared with admission. At 1-month follow-up after discharge, the SARA score had further decreased to 2, the PHQ-9 score to 3, the GAD-7 score to 0, and the craving VAS score remained 0, indicating sustained improvement in neurological symptoms, emotional symptoms, and craving.

## 3 Discussion

Unlike previously reported cases of tiletamine or tiletamine/zolazepam abuse, which mainly presented with acute intoxication-like manifestations such as involuntary movements, severe tremor, altered consciousness, hypotension, respiratory failure, and even death, the present case showed a relatively distinct clinical spectrum. The patient did not primarily present with marked disturbance of consciousness or cardiopulmonary impairment; rather, he developed subacute, progressively worsening dysarthria, choking on drinking, gait instability, broad-based gait, and tremor, accompanied by marked craving and impaired impulse control. Notably, these symptoms emerged during a period of frequent e-cigarette exposure and persisted after cessation, suggesting that the presentation was not merely a transient acute toxic reaction but more likely reflected persistent neuropsychiatric dysfunction following long-term repeated exposure. Thus, this case suggests that the clinical consequences of chronic inhalational tiletamine exposure via e-cigarettes may extend beyond the acute intoxication pattern recognized in previous reports and may also manifest as a subacute or chronic disorder characterized by persistent neurological involvement and dependence-related features.

From a neurological perspective,the patient’s manifestations, including gait instability, broad-based gait, impaired coordination testing, dysarthria, and choking on drinking, were overall more consistent with an ataxia-like clinical phenotype, suggesting possible involvement of cerebellar and/or brainstem-related functional networks. The precise pathophysiological basis remains unclear but may be related to disruption of central coordination networks, particularly cerebellar-brainstem circuits, after long-term tiletamine exposure. Previous animal studies of ketamine further suggest that NMDA receptor antagonists may interfere with the central coordination of orolingual-pharyngeal motor programs, which may provide an additional mechanistic clue for the dysarthria and choking observed in this case (16, 17). Ancillary findings also support a central rather than peripheral process. Brain MRI showed no obvious abnormalities, and electromyography did not reveal clear peripheral nerve or muscle damage. However, blink reflex testing demonstrated prolonged bilateral R2 and R2′ latencies. Previous studies have shown that R2/R2′ are polysynaptic late responses involving the trigeminal-brainstem-facial pathway, and abnormalities in these responses may indicate dysfunction of the late blink reflex arc, particularly in lower brainstem-related lesions (18–20). Taken together with the patient’s dysarthria, choking on drinking, and gait instability, these findings further support a central process, particularly involving brainstem-related pathways. Nevertheless, given the limited specificity of this electrophysiological marker and the lack of structural abnormalities on MRI, these results should be regarded as supportive rather than definitive localizing evidence.

In addition to persistent neurological involvement, the patient also exhibited relatively clear dependence-related features. Animal studies have shown that tiletamine itself can produce rewarding and reinforcing effects comparable to those of ketamine, possibly through modulation of the mesolimbic dopaminergic reward pathway after NMDA receptor blockade (21). This mechanism may partly explain the patient’s euphoric experience, repeated use, and persistent craving after cessation, and further suggests that tiletamine-containing e-cigarettes carry not only toxic risk but also considerable reinforcing and abuse potential.

The patient also showed prominent affective and behavioral abnormalities.Given the pharmacological similarity between tiletamine and ketamine, both acting primarily as NMDA receptor antagonists, previous findings on long-term ketamine misuse may provide indirect mechanistic clues for this case (22). and such affective abnormalities have been linked to altered functional connectivity in multiple emotion-regulation-related brain regions, including the subgenual anterior cingulate cortex (sgACC), orbitofrontal cortex (OFC), and ventromedial prefrontal cortex (vmPFC) (23). Although functional neuroimaging was not performed in this patient and similar network abnormalities therefore could not be directly confirmed, his PHQ-9 score indicated moderate-to-severe depressive symptoms, and, together with irritability, anxiety, and social withdrawal, suggests that chronic tiletamine exposure may also be associated with dysfunction of central emotion-regulation networks.

With regard to treatment and outcome, the patient’s neurological symptoms, emotional symptoms, and craving gradually improved after discontinuation of tiletamine-containing e-cigarettes and comprehensive supportive treatment. At discharge, the SARA score had decreased from 20 to 10, the PHQ-9 score from 15 to 6, the GAD-7 score from 9 to 3, and the craving VAS score from 6 to 0. At 1-month follow-up, the SARA score had further improved to 2, the PHQ-9 score had decreased to 3, the GAD-7 score to 0, and the craving VAS score remained 0. These changes suggest that the associated impairments may be at least partially reversible. However, it should be noted that the patient did not recover immediately after discontinuation; rather, he improved gradually with multidisciplinary comprehensive intervention. This suggests that tiletamine-related injury may not be confined to a transient acute intoxication process but may involve relatively persistent neuropsychiatric dysfunction requiring systematic treatment and follow-up. It also indicates that management of such patients should not be limited to simple abstinence or general supportive care, but should include integrated assessment and intervention targeting neurological symptoms, emotional and behavioral abnormalities, and substance-related problems.

From a clinical perspective, this case has important implications. In young patients presenting with gait instability, dysarthria, choking on drinking, tremor, or other unexplained neurological symptoms, especially when accompanied by emotional or behavioral changes, marked craving, or a history of e-cigarette use, clinicians should maintain a high level of suspicion for exposure to tiletamine or other novel psychoactive substances. Even when conventional structural neuroimaging and peripheral neurophysiological examinations do not reveal clear organic abnormalities, substance-related central nervous system dysfunction should not be readily excluded. Careful history-taking regarding e-cigarette use, suspected active ingredients, and patterns of substance use may be critical for identifying such cases.

From a public health perspective, this case should also be considered in the broader context of increasing e-cigarette use and the evolving delivery of novel psychoactive substances through vaping devices. Although population-level data specifically on tiletamine-containing e-cigarette use are not currently available, e-cigarette use is common among young adults. A recent online survey of Chinese adults aged 18–44 years reported that 12.9% of participants were current e-cigarette users, with higher prevalence among males and cigarette smokers (24). In addition, UNODC reported that, as of October 2025, 1,396 unique new psychoactive substances had been reported to the UNODC Early Warning Advisory by 153 countries and territories, and the annual number of unique NPS reported in 2024 reached a record high of 688 (25). UNODC has also highlighted that vaping products are increasingly being found to contain NPS and other psychoactive substances, including synthetic cannabinoids, ketamine, benzodiazepines, stimulants, opioids, and etomidate analogues (26, 27). These findings underscore the need for greater clinical awareness, toxicological surveillance, and regulatory attention to psychoactive substances delivered through vaping products, particularly among young users.

This case also has several limitations, the most critical of which is the absence of quantitative toxicological confirmation. Neither tiletamine testing in the patient’s biological samples nor mass spectrometric analysis of the e-cigarette cartridge or e-liquid was available; therefore, the exposure assessment relied mainly on the patient’s self-reported history. This absence of laboratory verification directly affects diagnostic certainty: the diagnosis cannot be considered toxicologically confirmed and should instead be interpreted as a clinically suspected case of tiletamine-containing e-cigarette–associated neuropsychiatric dysfunction. In addition, without chemical analysis of the cartridge or e-liquid, the possibility of other adulterants or co-existing psychoactive substances cannot be excluded. Potential alternative or contributing substances include etomidate or etomidate analogues, ketamine-like dissociatives, synthetic cannabinoids, benzodiazepines, stimulants, opioids, solvents, or other contaminants in illicit e-liquids.

However, several clinical features make tiletamine a plausible suspected contributor in this case. First, the patient reported that his main and most frequent exposure during the period of symptom emergence and progression was tiletamine-containing e-cigarettes. Second, although etomidate-containing e-cigarettes can cause neurological symptoms such as confusion, tremor, unsteady gait, syncope, and myoclonus, these presentations tend to be acute and may be accompanied by metabolic or endocrine abnormalities such as hypokalemia or adrenal dysfunction (4, 28). In reported etomidate-related e-cigarette cases, severe hypokalemia, lower-limb weakness, difficulty walking, respiratory difficulty, adrenal abnormalities, and improvement after potassium supplementation have been described (4). In contrast, the present patient had normal electrolytes and no clear evidence of acute cardiopulmonary depression or severe disturbance of consciousness during admission. Third, the persistence of dysarthria, gait instability, ataxia-like signs, emotional symptoms, and craving after cessation is more consistent with a subacute or chronic neuropsychiatric process than with a brief intoxication episode alone. Therefore, while other adulterants cannot be completely ruled out, the temporal relationship and clinical pattern support chronic tiletamine exposure as the most relevant suspected exposure. Future cases should include toxicological testing of both biological samples and e-cigarette liquids whenever possible.

In conclusion, this case suggests that suspected chronic exposure to tiletamine-containing e-cigarettes may be associated with clinical consequences extending beyond the acute intoxication pattern recognized in previous reports. In addition to persistent neurological involvement, such exposure may also be accompanied by marked craving and affective abnormalities. Although the absence of toxicological confirmation limits diagnostic and causal certainty, this case expands the currently recognized clinical spectrum of suspected tiletamine-containing e-cigarette abuse and highlights the need to consider novel psychoactive substance-related e-cigarette exposure in the differential diagnosis of young patients with otherwise unexplained neuropsychiatric symptoms.

## Data Availability

The original contributions presented in the study are included in the article/supplementary material. Further inquiries can be directed to the corresponding author.
